# Pan-Chromosome and Comparative Analysis of *Agrobacterium fabrum* Reveal Important Traits Concerning the Genetic Diversity, Evolutionary Dynamics, and Niche Adaptation of the Species

**DOI:** 10.1128/spectrum.02924-22

**Published:** 2023-02-28

**Authors:** Yuhui Du, Jinrong Zou, Zhiqiu Yin, Tingjian Chen

**Affiliations:** a MOE International Joint Research Laboratory on Synthetic Biology and Medicines, School of Biology and Biological Engineering, South China University of Technology, Guangzhou, People’s Republic of China; b Clinical Laboratory Department, Fifth Affiliated Hospital of Guangzhou Medical University, Guangzhou, People’s Republic of China; c National Engineering Research Center for Efficient Utilization of Soil and Fertilizer Resources, College of Resources and Environment, Shandong Agricultural University, Tai’an, People’s Republic of China; Tianjin University

**Keywords:** *Agrobacterium fabrum*, pan-chromosome, genetic diversity, evolutionary dynamics, genomic plasticity, pathogenicity, niche adaptation

## Abstract

Agrobacterium fabrum has been critical for the development of plant genetic engineering and agricultural biotechnology due to its ability to transform eukaryotic cells. However, the gene composition, evolutionary dynamics, and niche adaptation of this species is still unknown. Therefore, we established a comparative genomic analysis based on a pan-chromosome data set to evaluate the genetic diversity of *A. fabrum*. Here, 25 *A. fabrum* genomes were selected for analysis by core genome phylogeny combined with the average nucleotide identity (ANI), amino acid identity (AAI), and *in silico* DNA-DNA hybridization (DDH) values. An open pan-genome of *A. fabrum* exhibits genetic diversity with variable accessorial genes as evidenced by a consensus pan-genome of 12 representative genomes. The genomic plasticity of *A. fabrum* is apparent in its putative sequences for mobile genetic elements (MGEs), limited horizontal gene transfer barriers, and potentially horizontally transferred genes. The evolutionary constraints and functional enrichment in the pan-chromosome were measured by the Clusters of Orthologous Groups (COG) categories using eggNOG-mapper software, and the nonsynonymous/synonymous rate ratio (*dN*/*dS*) was determined using HYPHY software. Comparative analysis revealed significant differences in the functional enrichment and the degree of purifying selection between the core genome and non-core genome. We demonstrate that the core gene families undergo stronger purifying selection but have a significant bias to contain one or more positively selected sites. Furthermore, although they shared similar genetic diversity, we observed significant differences between chromosome 1 (Chr I) and the chromid in their functional features and evolutionary constraints. We demonstrate that putative genetic elements responsible for plant infection, ecological adaptation, and speciation represent the core genome, highlighting their importance in the adaptation of *A. fabrum* to plant-related niches. Our pan-chromosome analysis of *A. fabrum* provides comprehensive insights into the genetic properties, evolutionary patterns, and niche adaptation of the species.

**IMPORTANCE** *Agrobacterium* spp. live in diverse plant-associated niches such as soil, the rhizosphere, and vegetation, which are challenged by multiple stressors such as diverse energy sources, plant defenses, and microbial competition. They have evolved the ability to utilize diverse resources, escape plant defenses, and defeat competitors. However, the underlying genetic diversity and evolutionary dynamics of *Agrobacterium* spp. remain unexplored. We examined the phylogeny and pan-genome of *A. fabrum* to define intraspecies evolutionary relationships. Our results indicate an open pan-genome and numerous MGEs and horizontally transferred genes among *A. fabrum* genomes, reflecting the flexibility of the chromosomes and the potential for genetic exchange. Furthermore, we observed significant differences in the functional features and evolutionary constraints between the core and accessory genomes and between Chr I and the chromid, respectively.

## INTRODUCTION

The genus *Agrobacterium*, created by Conn et al. (1), is an alphaproteobacterium of the family *Rhizobiaceae*. It is robust and versatile tool used to transfer foreign genes into plants or fungi due to its ability to transform eukaryotic cells (2). Most members of the genus are primarily plant pathogens, causing either crown gall or hairy root disease, depending on the presence of a tumor-inducing Ti plasmid or a root-inducing Ri plasmid in their genome (3). The avirulent members of *Agrobacterium* without the Ti plasmid are common members of soil communities, and efficiently colonize the rhizospheres of a wide variety of plant hosts (4). They are also recognized as soil- and rhizosphere-adapted bacteria. *Agrobacterium* populations live in diverse ecological niches and thus face different habitat constraints. They have evolved the ability to adapt to diverse ecological niches, escape plant defenses, and outcompete coexisting bacteria. However, the genetic properties and genome evolution which favor the adaptation of *Agrobacterium* members to diverse niches remain largely unidentified.

Twenty-one species are currently recognized in the *Agrobacterium* genus (List of Prokaryotic names with Standing in Nomenclature [LSPN] database) (5). *Agrobacterium fabrum*, formerly known as Agrobacterium tumefaciens genomovar G8, belongs to the A. tumefaciens taxonomic complex (6, 7). *A. fabrum* C58 was the first bacterium of the genus subjected to genomic DNA sequencing due to its application in plant genetic engineering (8, 9). Analysis of the *A. fabrum* C58 genome revealed an unusual structure including a chromosome and chromid (10) of similar gene densities and divergent genomic properties (8). The *A. fabrum* chromid derives from a plasmid of a divergent origin (11); while the chromosome includes housekeeping and virulence genes (8, 9). Comparative genomic analysis of the *A. fabrum* chromosome and chromid identified higher plasticity in the former, with specific genetic elements differently localized in the genome, presumably in response to niche adaptation (12). We hypothesized that chromial and chromosomal structure differences between *A. fabrum* strains are evidence of adaptive evolution.

Twenty-eight publicly available genome sequences derived from *A. fabrum* were subjected to core genome phylogeny. Only 25 out of the 28 genome sequences clustered within the *A. fabrum* clade and were thus used for pan-genome determination. A precise pan-genome was defined with 12 reference genome sequences belonging to the main *A. fabrum* subclade. The genetic diversity of the species was defined by exploring the mobile genetic element (MGE) and horizontally transferred gene content. Adaptive evolution and genetic divergence were studied by determining the functional enrichment and selective pressure among the genomic chromosomes and chromids. Niche adaptation and virulence were studied by defining putative functions associated with carbohydrate utilization, biosynthesis of secondary metabolites, and virulence, such as the SpG8 loci, phytochromes, and the *mel* operon.

## RESULTS AND DISCUSSION

### Core genome phylogeny analysis of the *A. fabrum* genomic DNA sequences and other members of the genus.

**FIG 1:**
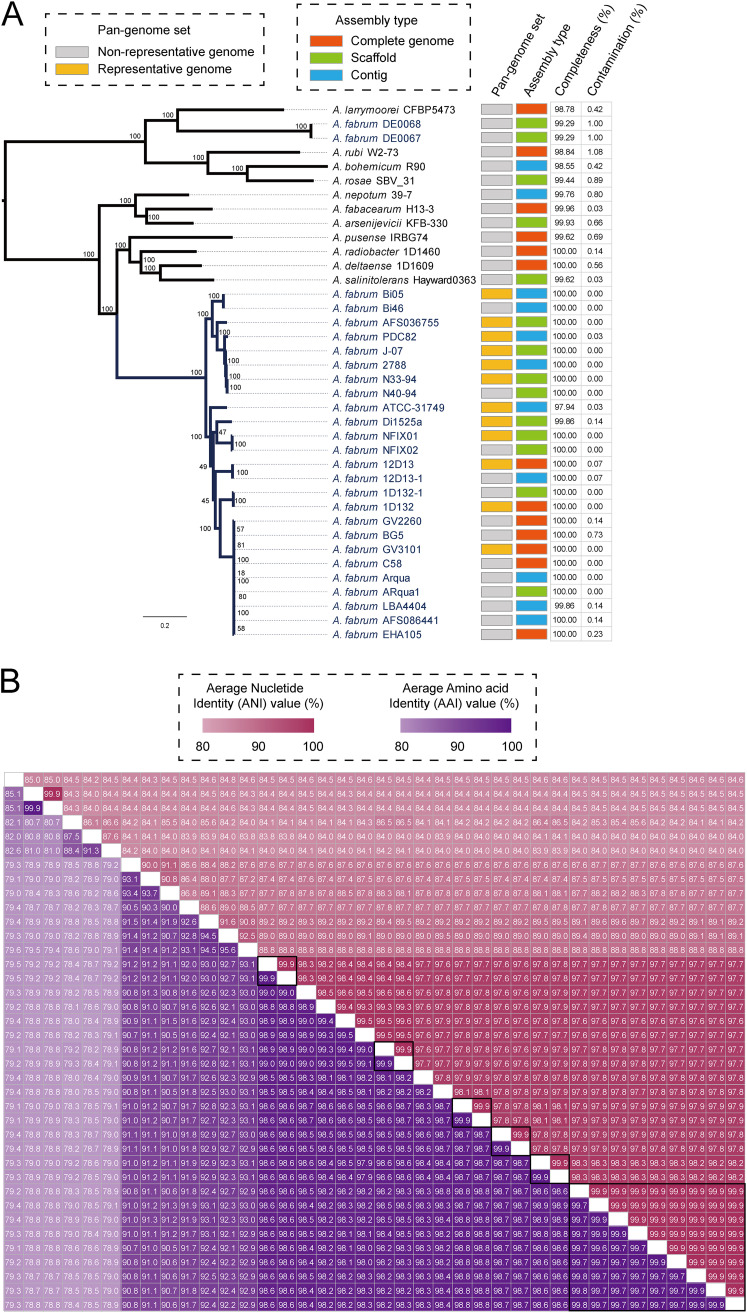
Genetic relatedness of *Agrobacterium* strains (*n *=* *38). (A) Core genome phylogeny. The maximum likelihood based on single-nucleotide polymorphisms (SNPs) across 2,252 single-copy core gene families shared by 38 *Agrobacterium* spp. genomes. Values on the primary nodes of the tree are bootstrap values (100 replicates). Color blocks next to the tree indicate the representative genomes used for pan-genome analysis and the assembly type of these genomes, respectively. Values next to the color blocks indicate the completeness (%) and contamination (%). (B) Heatmap showing pairwise average nucleotide identities (ANI, red upper section of the matrix) and amino acid identities (AAI, purple lower section of the matrix).

In the core genome tree, most (25/27) members of *A. fabrum* formed a monophyletic clade with a long branch length and were deeply nested within the *Agrobacterium* genus, confirming evolutionary divergence (Fig. 1A and Table S1). The phylogenetic tree in Fig. 1A identifies *A. salinitolerans*, *A. deltaense*, *A. radiobacter*, and *A. pusense* as neighboring species of *A. fabrum*. However, two strains with a failed taxonomy check, DE0067 and DE0068, were located outside the separate clade of *A. fabrum* in the core genome tree, exhibiting a low level of genetic relatedness to other *A. fabrum* members.

The average nucleotide identity (ANI) and average amino acid identity (AAI) were used to further measure the genetic relatedness between *Agrobacterium* strains. In the separate clade of *A. fabrum*, the members shared high ANI (>97.6%) and AAI (>97.9%) values with each other, above the 95% threshold value for species circumscription (13) (Fig. 1B), including PDC82, NFIX01, NFIX02, AFS036755, AFS086441, and LBA4404, originally identified as other species. The ANI and AAI values determined from comparisons between *A. fabrum* DE0067 and DE0068 and other members of *A. fabrum* genomes were 84.3% to 84.5% and 78.8% to 79.2%, respectively, confirming a classification outside the boundaries of the species. This observation was confirmed by the *in silico* DDH values for *A. fabrum* DE0067 and DE0068 against *A. fabrum* C58 of 22% each, well below the recommended 70% for species inclusivity (14). Thus, we propose the reclassification of *A. fabrum* DE0067 and DE0068 as another *Agrobacterium* species. In conclusion, the phylogeny analysis identified 25 genome sequences as pertaining to the *A. fabrum* species, out of the 28 originally considered for the study, for inclusion in subsequent pan-genome analysis.

### General information for the *A. fabrum* chromosomes.

As shown in Table 1, the chromosomal sizes of these *A. fabrum* genomes range from 4,833.2 to 4,974.1 Kb and contain 4,659 to 5,217 genes. The strains 12D13-1 and ATCC-31749 had the minimum and maximum genome sizes and gene contents, respectively. The average *A. fabrum* genome size, GC content, and gene number were 4,899.6 ± 29.3 Kb, 59.1 ± 0.08%, and 4,783.4 ± 99.5, respectively. *Agrobacterium* chromosome I (Chr I) is larger (about 2.8 Mb), carrying most essential protein-coding genes, whereas chromosome II (the chromid) is smaller (about 2.0 Mb) and carries a minority of essential genes (8, 9). The sizes of Chr I estimated in this study ranged from 2,825.3 (AFS086441) to 3,053.2 Kb (ATCC-31749), an average of 2,876.6 ± 52.01 Kb, with 59.3 ± 0.08% GC content. The gene number of Chr I varied from 2,843 (12D13-1) to 3,304 (ATCC-31749), an average of 2,934.6 ± 89.4 per chromosome. Meanwhile, for the chromid, the estimated sizes ranged from 1,920.9 (ATCC-31749) to 2,076.2 Kb (NFIX01) with an average of 2,023.1 ± 46.5 Kb and 59.3 ± 0.07% GC content. The chromid gene number ranged from 2,843 (12D13-1) to 3,304 (ATCC-31749), an average of 1,848.8 ± 52.04 per chromosome. Interestingly, despite possessing the largest genome (4,974.1 Kb) and Chr I (3,053.2 Kb) of the *A. fabrum* species, ATCC-31749 has the smallest chromid (1,920.9 Kb). The variability of the putative gene functions and gene number of the *A. fabrum* chromosome compared to its chromid confirms the divergence in origin.

**TABLE 1:** General chromosomal characteristics of the 25 Agrobacterium fabrum genomes included in this study^*a*^

Strain	Total chr			Chr I			Chromid		
	Length (Kb)	GC (%)	Gene no.	Length (Kb)	GC (%)	Gene no.	Length (Kb)	GC (%)	Gene no.
Bi05	4,890.948	59.3	4,731	2,923.068	59.3	2,966	1,967.88	59.2	1,765
Bi46	4,890.473	59.3	4,738	2,922.993	59.3	2,970	1,967.48	59.2	1,768
AFS036755	4,850.775	59.4	4,759	2,888.718	59.3	2,981	1,962.057	59.4	1,778
PDC82	4,946.854	59.3	4,791	2,953.585	59.3	3,007	1,993.269	59.4	1,784
J-07	4,895.908	59.3	4,757	2,914.578	59.3	2,964	1,981.33	59.4	1,793
2788	4,902.562	59.4	4,777	2,891.936	59.3	2,955	2,010.626	59.4	1,822
N33-94	4,881.621	59.3	4,744	2,869.927	59.3	2,916	2,011.694	59.2	1,828
N40-94	4,881.415	59.3	4,726	2,870.051	59.3	2,906	2,011.364	59.2	1,820
ATCC-31749	4,974.09	59.2	5,217	3,053.148	59.0	3,304	1,920.942	59.4	1,913
Di1525a	4,904.917	59.3	4,781	2,909.821	59.3	2,975	1,995.096	59.3	1,806
NFIX01	4,909.985	59.3	4,783	2,833.79	59.4	2,884	2,076.195	59.3	1,899
NFIX02	4,908.208	59.3	4,781	2,833.351	59.4	2,879	2,074.857	59.3	1,902
12D13	4,852.14	59.4	4,670	2,853.758	59.3	2,853	1,998.382	59.5	1,817
12D13-1	4,833.229	59.4	4,659	2,847.681	59.3	2,843	1,985.548	59.5	1,816
1D132-1	4,882.735	59.3	4,770	2,892.522	59.2	2,951	1,990.213	59.3	1,819
1D132	4,921.389	59.3	4,774	2,907.491	59.2	2,959	2,013.898	59.3	1,815
GV2260	4,917.156	59.3	4,782	2,841.579	59.4	2,886	2,075.577	59.3	1,896
BG5	4,921.631	59.3	4,882	2,865.988	59.3	2,964	2,055.643	59.3	1,918
GV3101	4,917.155	59.3	4,770	2,841.579	59.4	2,878	2,075.576	59.3	1,892
C58	4,917.157	59.3	4,779	2,841.58	59.4	2,887	2,075.577	59.3	1,892
Arqua	4,899.323	59.4	4,787	2,830.405	59.4	2,890	2,068.918	59.3	1,897
ARqua1	4,891.352	59.4	4,783	2,828.098	59.4	2,891	2,063.254	59.3	1,892
LBA4404	4,892.498	59.4	4,778	2,829.248	59.4	2,881	2,063.25	59.3	1,897
AFS086441	4,888.406	59.4	4,782	2,825.321	59.4	2,887	2,063.085	59.3	1,895
EHA105	4,919.214	59.3	4,785	2,843.716	59.4	2,888	2,075.498	59.3	1,897

a chr, chromosome.

### Pan-genome architecture of *A. fabrum*.

**FIG 2:**
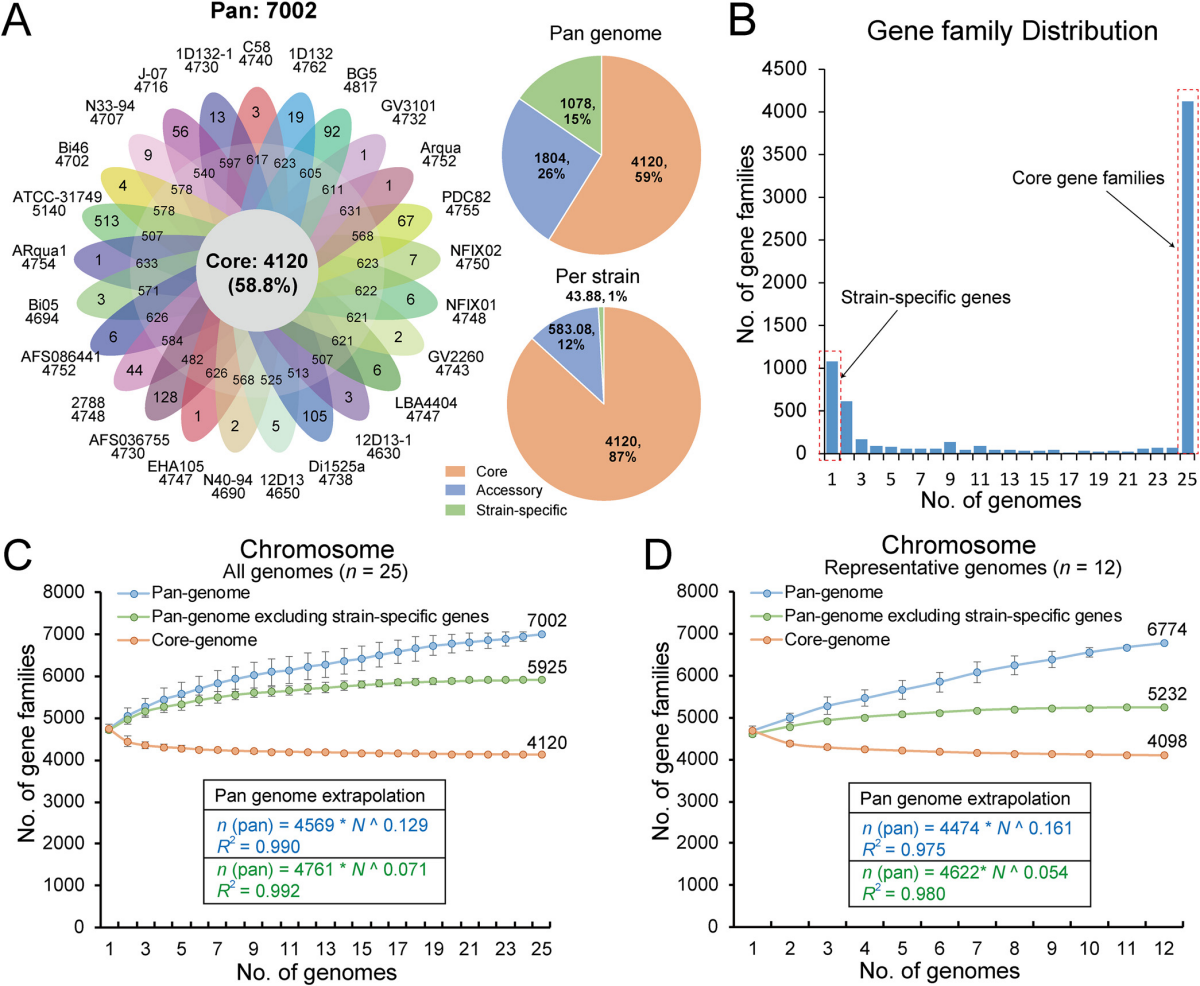
Pan-genome analysis of the *A. fabrum* genome. (A) Pan-genome of 25 *A. fabrum* genomes. Flower plot of 25 *A. fabrum* genomes showing the gene content of the core genome (center), the accessory genome (around the center), and strain-specific genes (petals). Pie charts show the percentages of core, accessory, and strain-specific gene families participating in the *A. fabrum* pan-genome. (B) Distribution of gene family sizes in the pan-genome generated from 25 *A. fabrum* genomes, indicating the number of strain-specific genes and core gene families. (C) Progressive curves for the core genome, pan-genome, and pan-genome excluding strain-specific genes were estimated for all 25 genomes. Curves show the downward trend of the core gene families and the upward trend of the pan-gene families with additional genomes. Deduced mathematical functions of pan-genome curves are shown inside the curves. (D) Progressive curves for the core, pan-genome, and pan-genome excluding strain-specific genes were estimated for a set of 12 representative genomes screened by the genetic relatedness of all 25 genomes.

A total of 7,002 homologous gene families were identified from a collection of all 25 *A. fabrum* genomes (Fig. 2A and Table S2). Among these, 4,120 (58.8%), which comprise the largest group, representing the core genome are present across all 25 genomes; 1,804 (25.8%) are present in at least one *A. fabrum* genome, representing the accessory genome; and the remaining 1,078 (15.4%) are only present in one genome, representing the strain-specific gene content (Fig. 2A). The numbers of accessory gene families in the *A. fabrum* genomes range from 172 to 383 gene families with an average of 334.0 ± 80.4 families. The incidence of strain-specific genes ranged from 10 to 272 with an average of 82.3 ± 97.0, suggesting highly divergent intraspecies genomes (15). As shown in Fig. 2B, most non-core gene families are not broadly distributed (≤2 genomes), indicating that the flexibility of the gene content is strain-specific.

To determine whether a pan-genome was open or closed (16), the accumulation curve for each genome added was determined and fit to Heaps’ power law function ($n={\mathrm{\kappa N}}^{\gamma }$), where *N* is the number of genomes and κ is a proportionality constant. The exponent γ indicates whether a pan-genome is open (γ ≥ 0) or closed (γ < 0). As shown in Fig. 2C, the pan-genome steadily increases in size with the addition of each additional genome, with an average of ~90 new genes are added to the pan-genome as each new *A. fabrum* genome is added. The *A. fabrum* pan-genome appears to be barely open (γ = 0.129), indicating that *A. fabrum* has a source gene pool from which to continuously acquire exogenous genetic elements. Additionally, it is notable that when strain-specific genes are excluded, a plateau appears in the pan-genome accumulation curve, suggesting that most undiscovered genes are not broadly distributed (17).

### Characteristic genetic diversity reflected by pan-genome of the representative data set.

A portion of the *A. fabrum* genomes included in this study exhibited close genetic relatedness (Fig. 1), which would lead to a bias in the pan-genome analysis. Therefore, based on the assembly quality, core genome tree, and genomic relatedness generated by ANI and AAI (Fig. 1), we selected a representative data set of 12 genomes, which represents an even distribution of *A. fabrum* genetic diversity. The pan-genome of 12 representative genomes was similar in size with 6,774 gene families, composed of 4,098 (60.5%) core gene families, 1,134 (16.7%) accessory gene families, and 1,542 (22.8%) strain-specific genes. The proportion of core gene families was similar in both the 25- and 12- genome data sets. However, the proportion of strain-specific genes (22.8%) in the representative data set was larger than that in the 25-genome data set (15.4%), representing greater genetic diversity among individual strains. The pan-genome of the representative data set was also open, with ~174 new genes added on average with each new genome added. The upward trend and growth exponent value (γ = 0.161) of this data set were greater than those of the 25-genome data set (Fig. 2D), reflecting greater genetic diversity (16).

### Similar genetic diversity in the Chr I and chromid pan-genomes.

**FIG 3:**
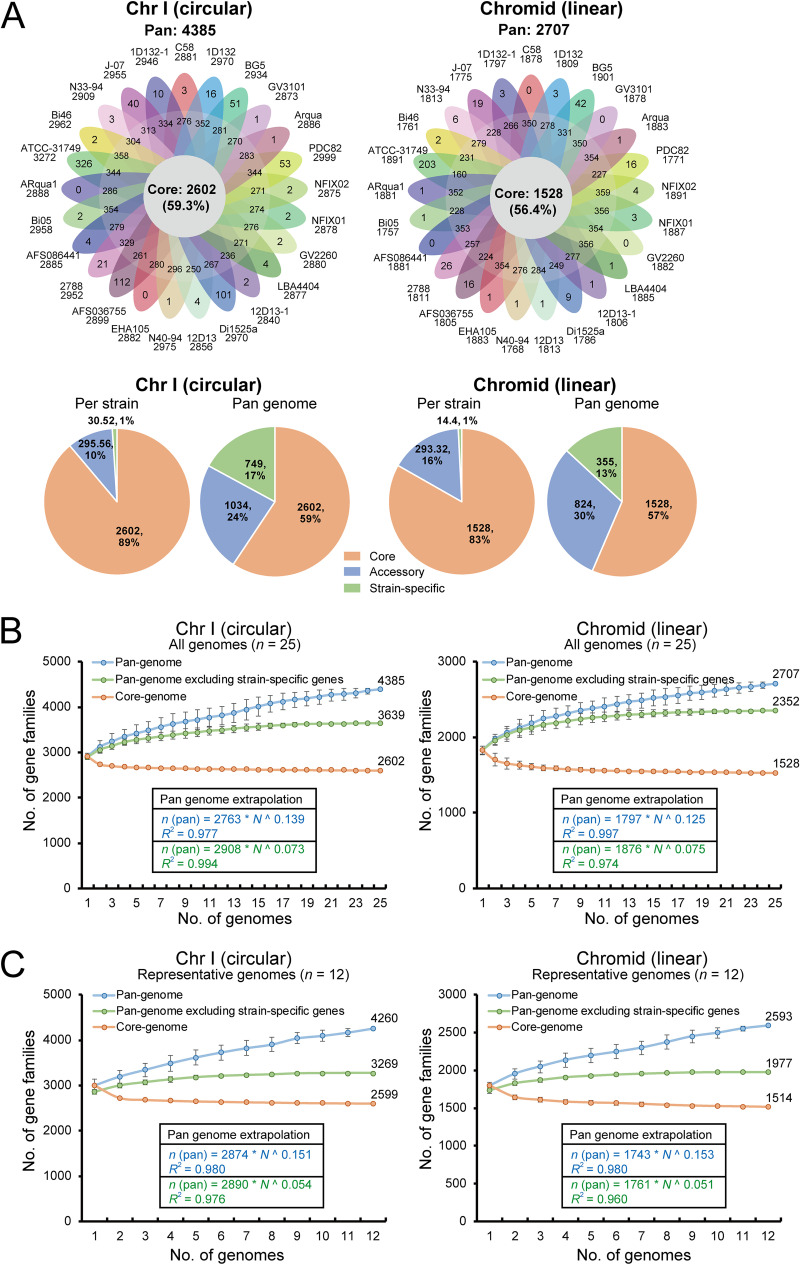
Pan-genome analysis of chromosome I (Chr I) and chromid. (A) Pan-genomes of Chr I and chromid for 25 *A. fabrum* genomes. Flower plot of Chr I and chromid of 25 *A. fabrum* genomes showing the gene content of core genome (center), accessory genome (around the center), and strain-specific genes (petals). Pie charts show the percentages of core, accessory, and strain-specific gene families participating in the Chr I and chromid pan-genomes. (B) Progressive curves for the core, pan-genome, and pan-genome excluding strain-specific genes were estimated for Chr I and the chromid of all 25 genomes. Curves show the downward trend of the core gene families and the upward trend of the pan-gene families with additional genomes. Deduced mathematical functions of pan-genome curves are shown inside the curves. (C) Progressive curves for the core genome, pan-genome, and pan-genome excluding strain-specific genes were estimated for Chr I and the chromid of 12 representative genomes.

For the pan-genome of all 25 genomes, 2,598 (63.1%) and 1,512 (36.7%) core gene families, 979 (54.3%) and 759 (42.1%) accessory gene families, and 718 (66.6%) and 360 (33.4%) strain-specific genes were detected in Chr I and the chromid (Fig. S1A), respectively. This indicated that the gene families in Chr I and the chromid were evenly distributed in each component of the pan-genome proportionally to the chromosome sizes. In addition, we identified 76 gene families shared by both chromosomes, including 10 core gene families and 66 accessory gene families (Fig. S1B). To evaluate the genetic diversity at the chromosome level, we carried out pan-genome analysis for Chr I and the chromid. The Chr I pan-genome of 25 genomes consisted of 4,385 gene families, including 2,602 (59.3%) core genes, 1,034 (23.6%) accessory genes, and 749 (17.1%) strain-specific genes (Fig. 3A). For the chromid, a total of 2,707 pan-genome gene families were identified, including 1,528 (56.4%) core genes, 824 (30.4%) accessory genes, and 355 (13.1%) strain-specific genes (Fig. 3A). The component proportions of the Chr I and chromid pan-genomes reflected similar genetic diversity between the two chromosomes.

Using Heaps’ power law function, the Chr I and chromid pan-genomes of 25 genomes were shown to be open, with positive exponent values (γ = 0.139 for Chr I; γ = 0.125 for chromid) (Fig. 3B). For the data set of 12 representative genomes, the Chr I and chromid pan-genomes were also open (Fig. 3C). They had similar upward trends and growth exponent values (γ = 0.151 for Chr I; γ = 0.153 for chromid), which also reflect similar genetic diversity. However, previous comparative genomic analysis of the C58 genome found that the linear chromosome exhibited higher plasticity and was much less conserved than the circular chromosome (12, 18). Other bacteria with multi-chromosome genomes, such as *Vibrio* spp., have greater genetic diversity in the chromid than in Chr I (19, 20). Thus, it is interesting that the Chr I and chromid pan-genomes of *A. fabrum* demonstrated similar genetic diversity.

### Genomic plasticity of the *A. fabrum* chromosomes characterized by tRNA, MGEs, and barriers to horizontal gene transfer.

**FIG 4:**
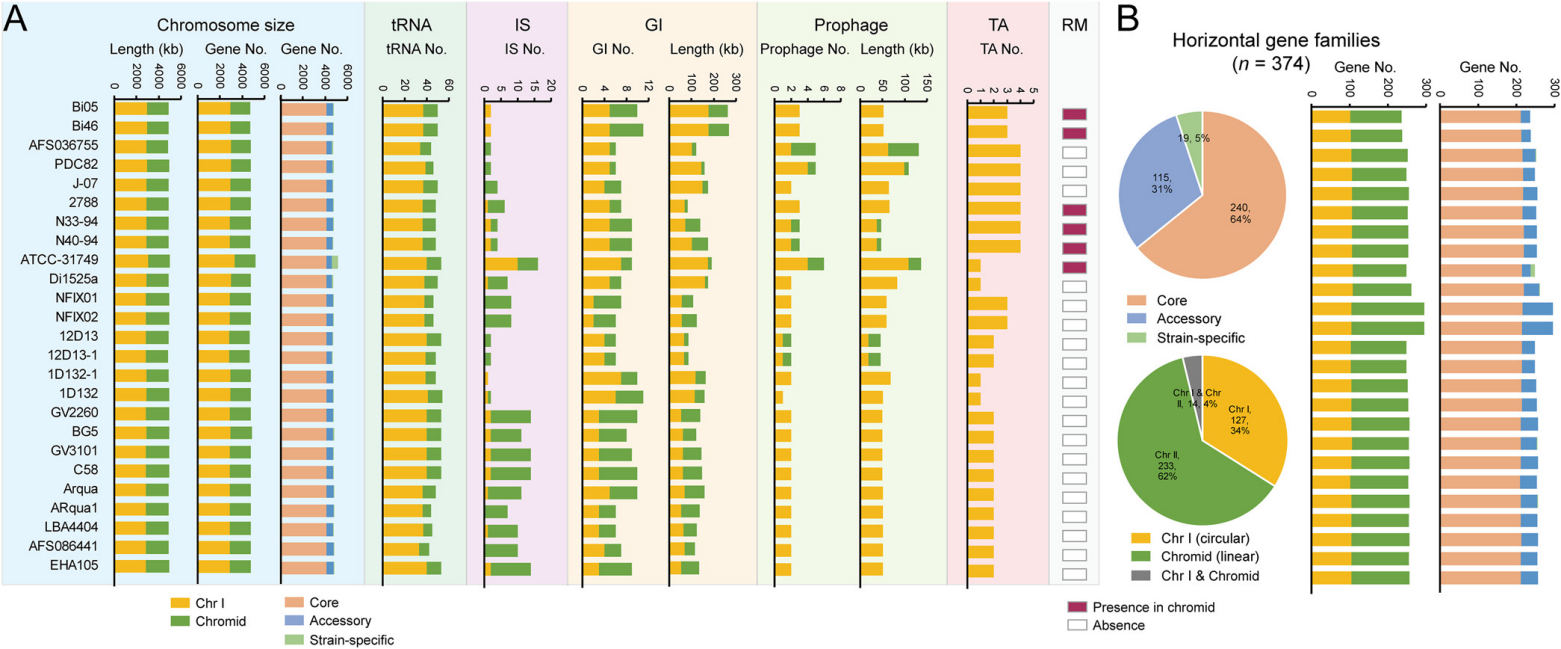
Mobile genetic elements (MGEs) and horizontal gene transfers (HGTs) in the *A. fabrum* genomes. (A) Distribution of chromosome size, tRNAs, MGEs, and multiple barriers to HGTs. (B) Distribution of potential horizontal gene families in the pan-genome, chromosome location, and each *A. fabrum* genome.

The open pan-genome with genetic diversity indicated the flexibility of the *A. fabrum* chromosomes. Consequently, we characterized the genomic plasticity of *A. fabrum* by evaluating tRNA loci, MGEs, and barriers to horizontal gene transfer (HGT). One *A. fabrum* genome contains an average of 49.04 ± 3.5 tRNA loci, including 37.9 ± 2.1 located on Chr I and 11.2 ± 2.2 located on the chromid (Fig. 4A). MGEs facilitate the acquisition of genes and contribute to the expansion of the bacterial gene pool (21, 22). In this study, we identified multiple types of MGEs, including insertion sequences (ISs), genomic islands (GIs), and prophages, which were heterogeneously distributed in the *A. fabrum* chromosomes (Fig. 4A). On average, one genome contains 7.1 ± 4.9 ISs, 8.1 ± 1.8 GIs spanning 147.3 ± 45.8 Kb in size, and 2.2 ± 1.0 prophages spanning 60.2 ± 28.4 Kb in size. Most ISs were located on the chromid in almost all *A. fabrum* genomes, except for ATCC-31749, which harbored 10 ISs on Chr I. GIs span almost 3.0% of the genome; 4.2 ± 1.4 (94.1 ± 44.05 Kb in size) and 3.8 ± 1.8 (53.2 ± 28.5 Kb in size) GIs were detected in Chr I and the chromid. For prophages, the number (2.2 ± 0.7) and covering regions (53.02 ± 22.8 Kb) of prophages of Chr I were more than those of the chromid (0.4 ± 0.8, spanning 7.2 ± 15.8 Kb in size). Our results indicate that the heterogeneous distribution of these MGEs contributes to the strain-specific genetic diversity of *A. fabrum*.

Several genetic elements known as barriers to HGT, such as the toxin/antitoxin (TA) system, the restriction-modification (RM) system, and clustered regularly interspaced short palindromic repeat (CRISPR), have been proposed to defend microbes against recurrent bacteriophage and plasmid infection and prevent foreign DNA uptake (23, 24). In this study, we successfully identified TA systems and RM systems in the *A. fabrum* genomes (Fig. 4A). All identified TA operons are heterogeneously distributed on Chr I. Six strains (Bi05, Bi46, 2788, N33-94, N40-94, and ATCC-31749) harbored one RM system in the chromid. No CRISPR was identified. The existence of these barriers to HGT is likely associated with the genome stability of *A. fabrum*. Overall, the distributions of tRNA, MGEs, and HGT barriers appear to vary with the genomic sizes and gene contents of some *A. fabrum* strains. For example, strain ATCC-31749, which has the largest chromosome (4,974.1 Kb) and highest gene content (5,217 genes, including 513 strain-specific genes), harbors a large number of tRNA loci (*n *=* *53, max 54), the most ISs (*n *=* *16), the largest region of prophages (*n *=* *6; 136.6 Kb), and the fewest TA operons (*n *=* *1). In contrast, the strain with the smallest genomic size (4,833.2 Kb, 4659 genes), 12D13-1, had only a few MGEs, which contained the fewest GIs (*n *=* *6, 85.9 kb in size), the smallest region of prophages (*n *=* *2, 45.9 kb), and only two ISs. Overall, the tRNA loci, numerous MGEs, and small number of HGT barriers heterogeneously distributed in the *A. fabrum* genomes contribute to their genomic diversity and can be major drivers of HGT and *A. fabrum* strain-specific evolution.

### Horizontal gene families in *A. fabrum* genomes, particularly those in the chromid.

HGT is the major driver of bacterial genetic diversity (25, 26). We identified 374 potential horizontal gene families in the *A. fabrum* genomes, including 240 (64.2%; 214.5 ± 2.8 per genome) core genes, 115 (30.4%; 40.2 ± 13.7 per genome) accessory genes, and 19 (5.1%; 0.8 ± 2.4 per genome) strain-specific genes (Fig. 4B and Table S3). This indicated that HGT occurring in the core genome may confer *A. fabrum* species-specific properties during the speciation process. Notably, many more horizontal gene families were located on the chromid. A total of 233 (62.3%; 150.8 ± 13.3 per genome) horizontal gene families were on the chromid, more than the 127 (34.0%; 104.8 ± 1.7 per genome) horizontal gene families on Chr I, indicating that HGT more significantly drives the genetic evolution of the chromid. Based on their Clusters of Orthologous Groups (COG) assignments, these horizontal gene families were involved in a variety of functional categories, including, mainly, “S: Function unknown” (*n *=* *65; 17.4%), “P: Inorganic ion transport and metabolism” (*n *=* *56; 15.0%), “K: Transcription” (*n *=* *38; 10.2%), and “E: Amino acid transport and metabolism” (*n *=* *34; 9.1%) (Fig. S2). HGT occurs at a relatively high rate in the accessory genome and has a disproportionate effect on strain adaptation in nature (27). Hence, these novel genetic properties driven by HGT may promote the adaptation of *A. fabrum* to diverse niches.

### Enrichment analysis revealed the functional divergence of the components of the pan-genome.

**FIG 5:**
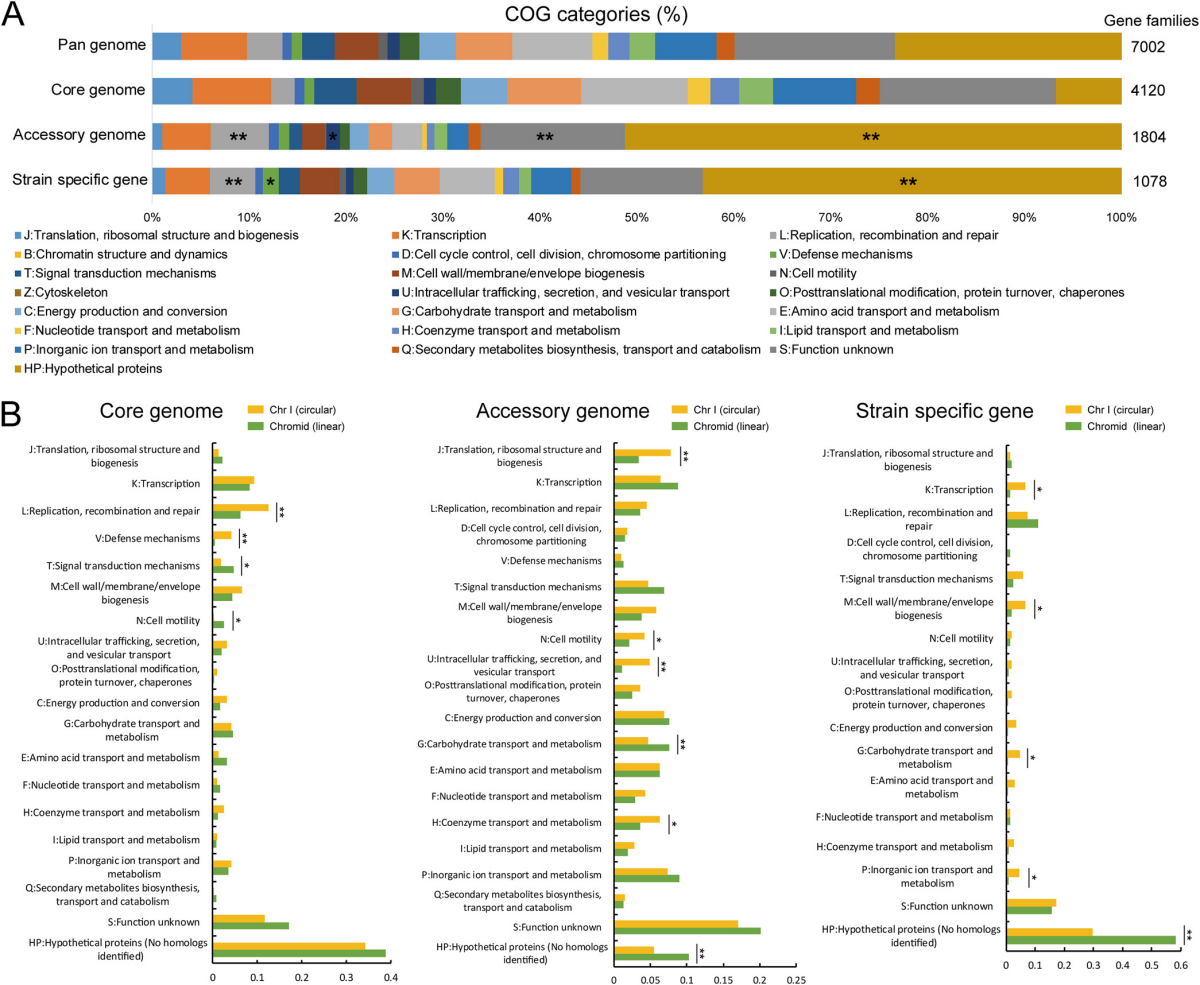
Comparison of functional enrichment for pan-genome. (A) Distribution of Clusters of Orthologous Groups (COG) functional categories for the pan-genome, core genome, accessory genome, and strain-specific genes, respectively. *, Fisher’s exact test *P* < 0.05; **, *P* < 0.01. (B) Comparison of COG functional categories for pan-genome, core genome, accessory genome, and strain-specific genes between Chr I and the chromid, respectively. *, *t* test *P* < 0.05; **, *P* < 0.01.

In this study, the gene families not annotated with COG functional categories were defined as “HP: Hypothetical proteins.” We observed functional divergence between different components of the pan-genome (Fig. 5A). The gene families assigned to “L: Replication, recombination and repair,” “HP: Hypothetical proteins,” “S: Function unknown” (Fisher’s exact test, *P* < 0.01), and “U: Intracellular trafficking, secretion, and vesicular transport” (Fisher’s exact test, *P* < 0.05) were significantly enriched in the accessory genome. Meanwhile, the strain-specific genes were significantly associated with multiple functional categories, including “L: Replication, recombination and repair,” “HP: Hypothetical proteins” (Fisher’s exact test, *P* < 0.01), and “V: Defense mechanisms” (Fisher’s exact test, *P* < 0.05) (Fig. 5A). These results indicated that the potential HGT events mainly occurred in the non-core genome, promoting the genetic diversity of the *A. fabrum* pan-genome.

A previous study revealed that the genes involved in essential processes are significantly overrepresented on Chr I (8). Here, to determine the functional divergence between Chr I and the chromid, we compared the functional categories of the core, accessory, and strain-specific gene families at the chromosome level. As shown in Fig. 5B, significant differences in the prevalence of functional categories were observed in different components of the pan-genome. For the core genome, Chr I harbored higher percentages of gene families involved in “L: Replication, recombination and repair” and “V: Defense mechanisms” (*t* test, *P* < 0.01), whereas the chromid core gene families were enriched in “T: Signal transduction mechanisms” and “N: Cell motility” (*t* test, *P* < 0.05). The Chr I accessory gene families were significantly associated with “J: Translation, ribosomal structure, and biogenesis,” “U: Intracellular trafficking, secretion, and vesicular transport” (*t* test, *P* < 0.01), “N: Cell motility,” and “H: Coenzyme transport and metabolism” (*t* test, *P* < 0.05) (Fig. 5B). Meanwhile, gene families assigned to “G: Carbohydrate transport and metabolism” and “HP: Hypothetical proteins” were prominently prevalent in the chromid accessory genome (*t* test, *P* < 0.01). The Chr I strain-specific genes were significantly involved in “K: Transcription,” “M: Cell wall/membrane/envelope biogenesis,” “G: Carbohydrate transport and metabolism,” and “P: Inorganic ion transport and metabolism” (*t* test, *P* < 0.05). Significantly, the chromid had a high proportion of strain-specific genes assigned to “HP: Hypothetical proteins” (*t* test, *P* < 0.01). These unknown functional genes with limited distribution contributed to the genetic diversity of the chromid, and their biological roles require further investigation.

### The difference of natural selection in the pan-genome demonstrated by comparative analysis of selective pressure.

**FIG 6:**
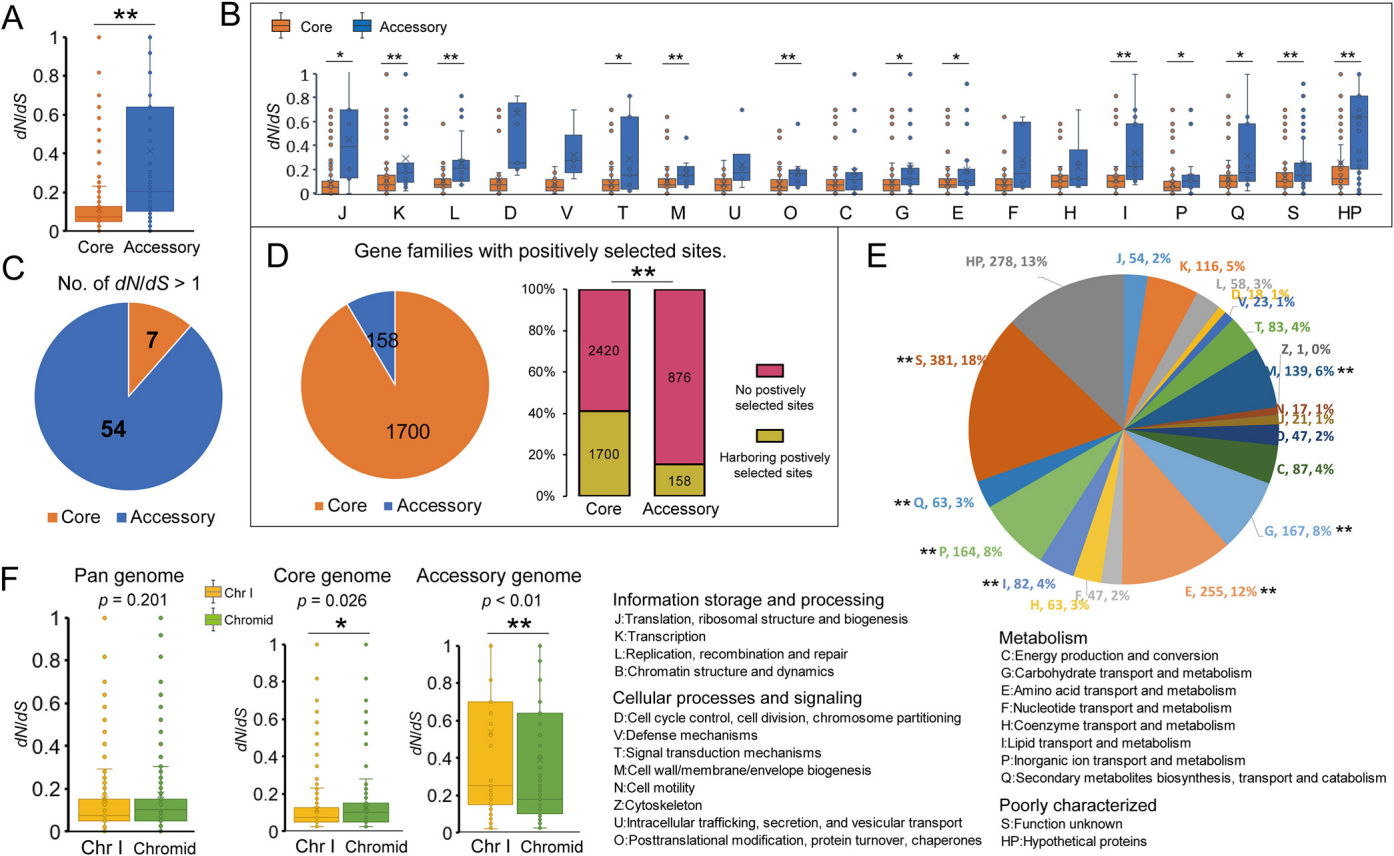
Analysis of selective pressure operated on the *A. fabrum* pan-genome. (A) Comparisons of the nonsynonymous/synonymous rate ratios (*dN*/*dS*) of gene families between the core and accessory genomes. **, *t* test *P* < 0.01. (B) Comparisons of the *dN*/*dS* ratios of gene families between the core genome and accessory genome in COG functional categories. *, *t* test *P* < 0.05; **, *P* < 0.01. (C) Distribution of positively selected gene families (*dN*/*dS *>* *1) in the pan-genome. (D) Analysis of positively selected sites within the gene families of the pan-genome. Pie chart shows distribution of gene families harboring positively selected sites. Diagram represents the relationship of gene families harboring positively selected sites between the core and accessory genomes based on chi-squared proportions tests. **, χ^2^ test *P* < 0.01. (E) Distribution of COG functional categories for gene families harboring positively selected sites. (F) Comparisons of the *dN*/*dS* ratios of gene families between Chr I and chromid in the pan-genome, core genome, and accessory genome, respectively. *, *t* test *P* < 0.05; **, *P*  < 0.01.

To explore how natural selection shapes the genetic properties of the *A. fabrum* pan-genome, we performed a codon-level analysis of natural selection measured by the nonsynonymous/synonymous rate ratio (*dN*/*dS*) on the 4,120 core gene families and 1,034 accessory gene families which were present in at least four *A. fabrum* genomes. Most gene families (average *dN*/*dS *=* *0.17 ± 0.26) evolved at low evolutionary rates, exhibiting a predominant action of purifying selection in the *A. fabrum* pan-genome. Obviously, the core gene families (average *dN*/*dS *=* *0.12 ± 0.15) have been under significantly stronger purifying selection than the accessory gene families (average *dN*/*dS *=* *0.44 ± 0.49 [*t* test, *P* < 0.01]) (Fig. 6A), indicating that the core genomes have a stronger tendency to keep their functions. The stronger evolutionary constraints of core gene families were also observed in the majority of functional categories (Fig. 6B). A total of 63 gene families were identified as positively selected (*dN/dS *>* *1), including 7 core gene families and 54 accessory gene families (Fig. 6C). Most of these gene families undergoing positive selection encoded hypothetical proteins, in addition to two genes that encoded transcriptional regulator (accessory gene family 242) and GNAT family acetyltransferase (accessory gene family 403), respectively (Table S4).

Although the entire coding regions were affected by the purifying selection, we identified numerous gene families (*n *=* *1m858) containing one or more codon sites which had significant evidence of positive selection (posterior probability ≥ 0.9) (Table S5). Among these, 1,700 (91.5%) represent the core gene families and the remaining 158 (8.5%) represent the accessory gene families (Fig. 6D). Interestingly, 41.3% (*n *=* *1700) of the 4,120 core gene families were found to have a signal of positive selection compared to 15.3% (*n *=* *158) of the 1,034 accessory gene families (chi-squared test, χ^2^ = 242.03, df = 1, *P* < 0.0001), indicating that there is a significant bias for core gene families to contain positively selected sites, even though the entire coding regions of these core gene families were constrained by stronger purifying selection. This bias is presumably because the evolutionary constraints of the core gene families are important for the species’ basic function and positively selected mutations in conserved gene families are permitted for gentle adaptation to diverse niches. Core genes comprising the backbone of bacterial genomes are not subject to frequent horizontal transfer and generally are not thought to contribute to adaptive evolution (28). However, the high frequency of positively selected mutations in *A. fabrum* core genome might also reflect the possibility that the evolving proteins have structural and functional constraints on residues capable of responding to natural selection, highlighting the potential role of the core genome in bacterial adaptive evolution. Furthermore, these gene families with positively selected sites were significantly involved in several metabolism categories, including “G: Carbohydrate transport and metabolism,” “E: Amino acid transport and metabolism,” “P: Inorganic ion transport and metabolism,” “I: Lipid transport and metabolism,” and “Q: Secondary metabolites biosynthesis, transport and catabolism” (Fisher’s exact test *P* < 0.05) (Fig. 6E). High metabolic flexibility in the open pan-genome is usually associated with the occupation of multiple niches (29, 30). It can be inferred that the potential variations driven by positive selection in metabolic properties seemed to reflect niche adaptation of *A. fabrum*.

### Differences in evolutionary signatures between Chr I and the chromid.

Considering the unusual chromosomal structure of *A. fabrum*, we then searched for differences in evolutionary signatures between Chr I and chromid. Overall, the Chr I gene families (average *dN*/*dS *=* *0.15 ± 0.26) have experienced a similar degree of selective pressure as the chromid gene families (average *dN*/*dS *=* *0.17 ± 0.27) (*t* test, *P* = 0.201) (Fig. 6F). Several studies have observed different evolutionary rates for each chromosome in a multipartite genome (19). The substitution rate of the secondary chromosome (chromid) of *Burkholderia* and *Vibrio* is higher than that of the chromosome, whereas purifying selection is weaker on the chromid (31). However, in *A. fabrum*, Chr I and the chromid have similar degrees of purifying selection. Interestingly, there are significant differences in evolutionary signatures between Chr I and the chromid in the core and accessory genome, respectively. As shown in Fig. 6F, the Chr I core gene families (average *dN*/*dS *=* *0.12 ± 0.14) have undergone significantly stronger purifying selection than those of the chromid (average *dN*/*dS *=* *0.13 ± 0.17) (*t* test, *P* = 0.026). For example, the evolutionary constraints of the Chr I core gene families involved in “L: Replication, recombination and repair,” “C: Energy production and conversion,” “I: Lipid transport and metabolism,” and “S: Function unknown” are significantly stronger than those of the chromid core gene families (*t* test, *P* < 0.05) (Fig. S3). The genes related to essential processes are found to be significantly overrepresented on C58 Chr I (8). We hypothesize that nomadic bacteria can modify chromid-borne genes as needed (32). By this assumption, weaker purifying selection should operate on chromids because of their lower necessity or usage (31). Hence, the Chr I core gene families of *A. fabrum*, essential for cell viability, understandably exhibited stronger evolutionary constraints than the chromid core gene families. In contrast, for the accessory genome, the purifying selection operating on chromid (average *dN*/*dS *=* *0.39 ± 0.47) was significantly stronger than that on Chr I (average *dN*/*dS *=* *0.54 ± 0.54) (*t* test, *P* < 0.01), which mainly involved the gene families associated with “E: Amino acid transport and metabolism” (*t* test, *P* < 0.01) (Fig. 6F and Fig. S3). Generally, the chromid accessory genomes in bacteria experience weakly selective constraints and evolve more rapidly, likely because they are used less frequently (31). Why accessory gene families evolve slower on the chromid in *A. fabrum* deserves further study. It is possible that the accessory genome on the *A. fabrum* chromid contains genes which are important for niche adaptation and therefore need to be conserved by evolutionary constraints.

### Carbohydrate-active enzymes and secondary metabolite biosynthesis gene clusters in *A. fabrum*.

**FIG 7:**
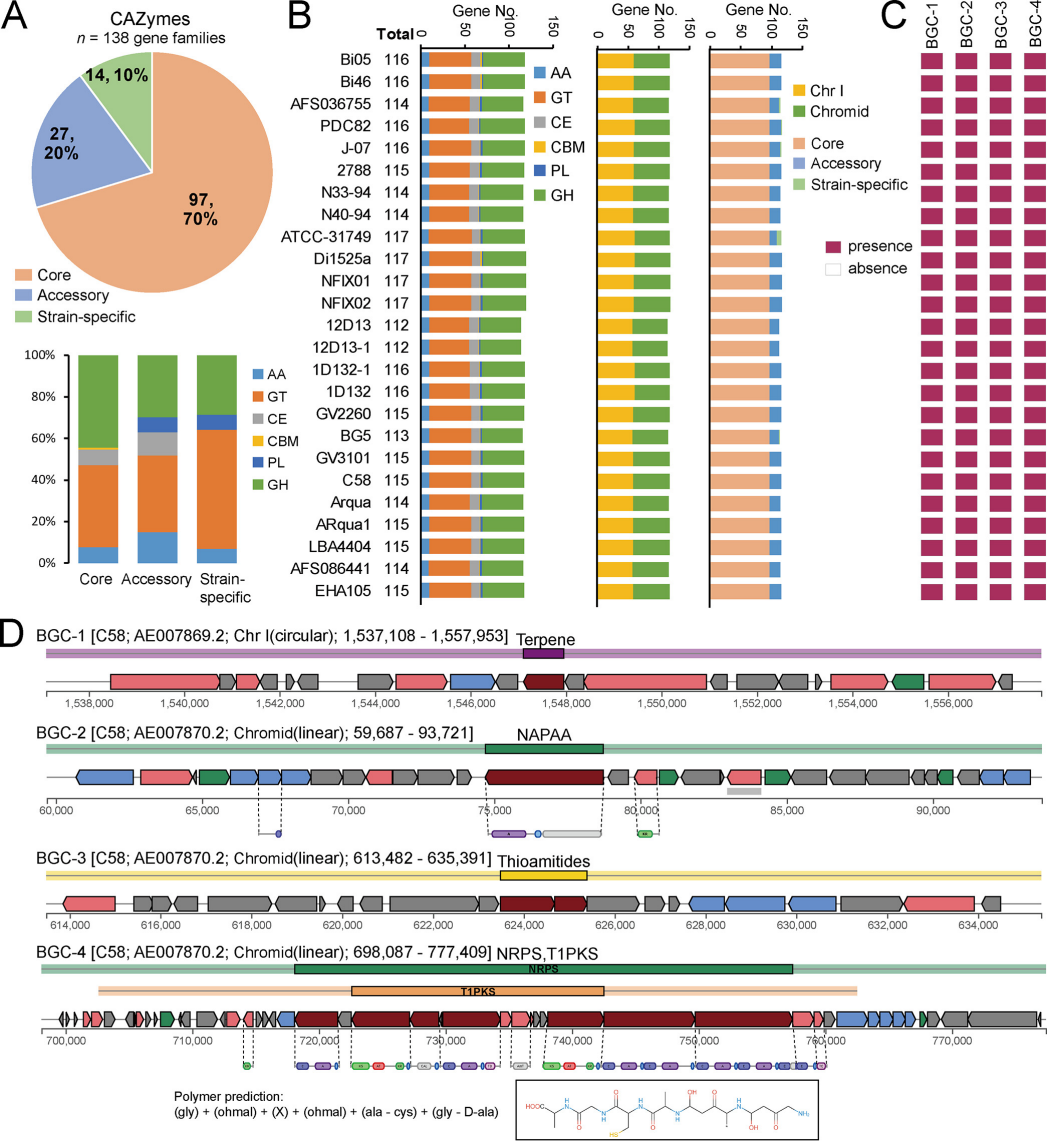
Carbohydrate active enzymes (CAZymes) and secondary metabolism elements in all 25 *A. fabrum* genomes. (A) Pie chart shows distribution of CAZymes in the pan-genome. Diagram represents the relationship of CAZymes between the core genome, accessory genome, and strain-species genes. (B) Distribution and composition of CAZymes in each *A. fabrum* genome. (C) Genetic organization of the biosynthesis gene clusters for secondary metabolite. Inset box shows the predicted monomer structure for nonribosomal peptide synthetase (NRPS, BGC-4) related secondary metabolite. (D) Distribution of the biosynthesis gene clusters (BGCs) for secondary metabolite.

The complex carbohydrates of diverse niches, including soil, rhizosphere, and plants, may be the main nutrient sources of *A. fabrum*. Carbohydrate-active enzymes (CAZymes) are the most important enzymes for complex carbohydrate metabolism (33). A total of 138 CAZyme-encoding gene families were identified in the pan-genome. Among these, the largest group of 97 (70.3%) represented the core genome and the remaining 27 (19.6%) and 14 (10.1%) represented accessory genome and strain-specific gene content, respectively (Fig. 7A). On average, one genome contained 97.0 ± 0.0 core, 17.4 ± 2.0 accessory, and 0.6 ± 1.7 strain-specific CAZyme-encoding genes. These CAZymes included auxiliary activities (AAs), glycosyltransferases (GTs), carbohydrate esterases (CEs), carbohydrate-binding molecules (CBMs), polysaccharide lyases (PLs), and glycoside hydrolases (GHs). An average genome contains 115.0 ± 1.4 CAZyme-encoding genes which encode 9.9 ± 0.3 AAs, 46.6 ± 1.0 GTs, 10.0 ± 0.4 CEs, 1.0 ± 0.0 CBMs, 2.0 ± 0.033 PLs, and 47.6 ± 1.0 GHs (Fig. 7B). About half of these are located on Chr I (58.5 ± 1.0), and the other half are on the chromid (58.5 ± 0.7). These CAZymes may be vital in the biosynthesis and degradation of various biomolecules for *A. fabrum* to promote adaptation to diverse environments. Additionally, *A. fabrum* may employ some of these CAZymes to penetrate into the plant cell wall for the successful establishment of colonization and infection.

We also identified four potential biosynthesis gene clusters (BGCs) associated with secondary metabolite synthesis, including terpene (BGC-1), non-alpha polyamino acids (BGC-2), thioamitides (BGC-3), and nonribosomal peptide synthetase (NRPS, BGC-4) (Fig. 7C). These BGCs were present in the core genome, representing a general property of *A. fabrum* (Fig. 7D). Among these, BGC-1 was located on Chr I, and the remaining BGC-2 to -4 were located on the chromid. Furthermore, we searched the homology of these BGCs in the antiSMASH database (34) and found that they showed no similarities to any well-known BGCs present in antiSMASH. Hence, the potential biological functions of these cryptic BGCs in *A. fabrum* require further exploration.

### Genotypic and phenotypic profiles of virulence in the *A. fabrum* chromosomes.

**FIG 8:**
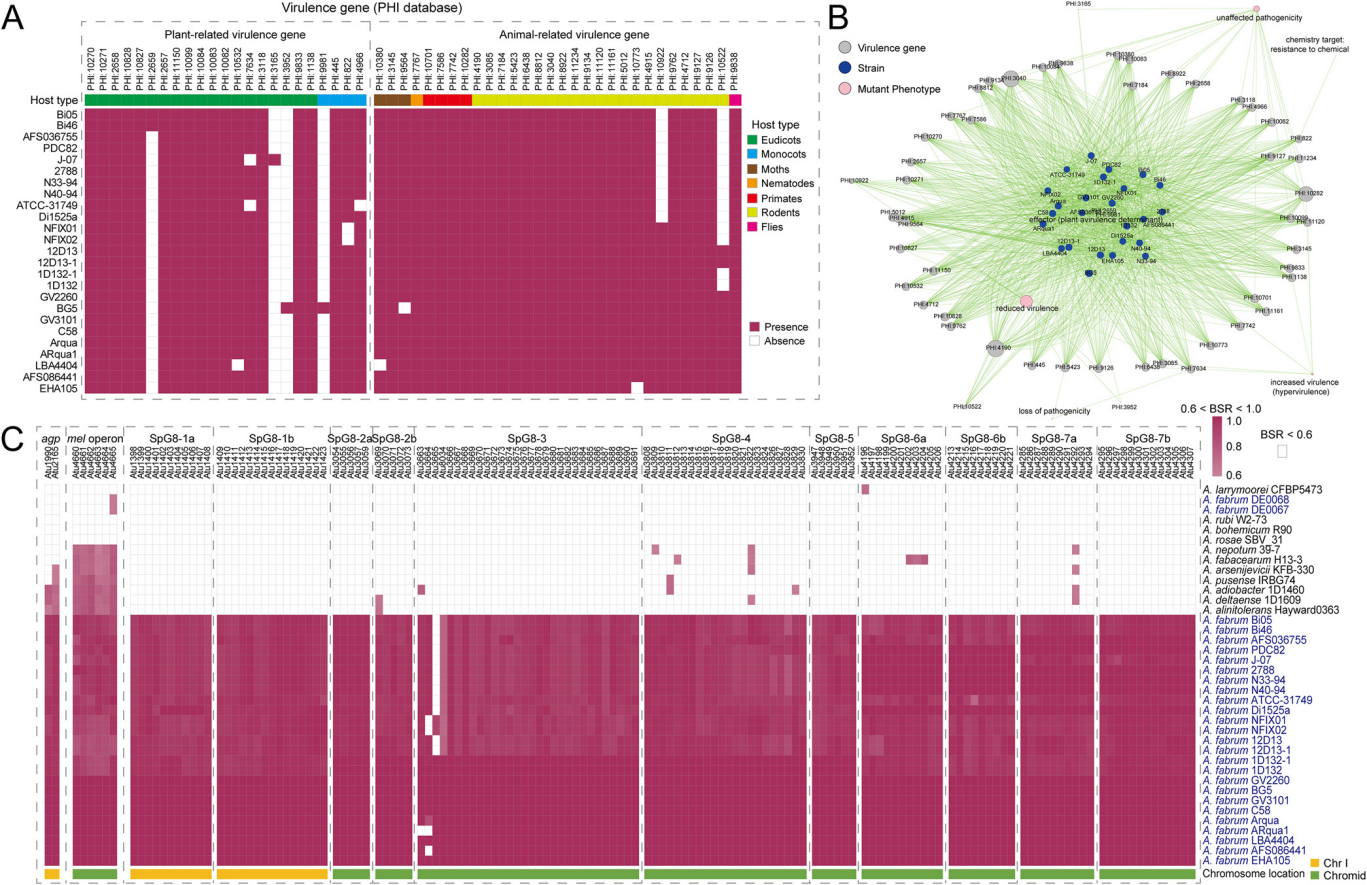
Distribution of virulence genes and key genetic elements in the *Agrobacterium* genomes. (A) Heatmap of the distribution of virulence and resistance genes in 25 *A. fabrum* genomes. Maroon block indicates presence of a gene, white block indicates absence. (B) Relationship network for different strains based on virulence genes and mutant phenotypes. (C) Heatmap of the distribution of *agp* gene, *mel* operon, and SpG8-1a to -7b in 38 *Agrobacterium* genomes. Color coding for blocks is based on the BLAST score ratios (BSR) calculated when the genomic data were screened against the reference genes. Genes with a BSR score of <0.6 were considered absent and are colored white.

Previous studies have found several virulence-related genes in the chromosome region of C58 (8, 9). In this study, a total of 53 gene families were found to match with virulence genes in the PHI-base database (Fig. 8A and Table S6). These virulence genes were predicted to have virulence-related phenotypic characteristics by mutation experiments (35). The dominant mutant phenotypes of these genes belonged to “reduced virulence” (*n *=* *37, 69.8%) (Fig. 8B), indicating that most of them were associated with determining the severity spectrum of infection, defined by virulence rather than the cause of infection as implied by the term pathogenicity. Most virulence-related gene families (*n *=* *47; 88.7%) represented common properties (present in more than 20 strains) of *A. fabrum*. The remaining 6 (11.3%) were sporadically distributed in the *A. fabrum* genomes (Fig. 8A). In addition, 23 identified virulence genes were related to plant hosts, including eudicots (*n *=* *18) and monocots (*n *=* *5). These plant-related virulence genes were associated with multiple processes such as infection, crown gall, tumor, soft rot, black rot, blackleg disease, bacterial speck, bacterial leaf blight, Fusarium ear blight, *Septoria* leaf blotch, *Septoria tritici* blotch, and leaf spot (see Table S6), indicating that virulence-related genes in the chromosome might play a vital role in *A. fabrum* pathogenicity.

One previous study found numerous orthologs of animal virulence genes in the C58 genome (9). In this study, more than half of the virulence genes identified (*n *=* *30) were related to animal hosts (rodents, 21; primates, 4; moths, 3; nematodes, 1; flies, 1). These virulence genes were involved in several diseases, including ovine brucellosis, brucellosis, nosocomial infection, food poisoning, and so on (Table S6). We need to further clarify the molecular roles which these identified virulence-related genes play in pathogenicity, especially in animal diseases. The safety of the genetically tractable *Agrobacterium* system also deserves further attention.

### Key genetic characteristics of the *A. fabrum* pan-genome.

Previous studies have analyzed the gene repertoire in *A. fabrum*, which contributes to plant infection, ecological adaptation, and speciation (12, 36, 37). Here, we examined these genetic elements in the *A. fabrum* pan-genome. As shown in Fig. 8C, seven known *A. fabrum*-specific genetic loci, SpG8-1 to -7, were found to be conserved in all 25 *A. fabrum* genomes and absent in other *Agrobacterium* spp. genomes. This result further supports these genomic regions as species-specific genetic traits, as identified by Lassalle et al. (12, 38). These SpG8 loci were associated with several biological functions, including metabolite catabolism (SpG8-1a, SpG8-1b, SpG8-4, and SpG8-5), secreted metabolite production (SpG8-2a and SpG8-3), detoxification (SpG8-6), and environmental sensing (SpG8-7), which were general properties of *A. fabrum*. We also found that two copies of phytochrome-encoding genes (*agp1* and *agp2*) were present in the core genome and located on Chr I. These phytochromes might promote plant infection by participating in the regulation of conjugation, plant infection, and DNA transfer into plants (36). Additionally, the *mel* operon was identified to be present on the chromid in all *A. fabrum* genomes. Meyer et al. (37) found that MelB protein (periplasmic-binding proteins) and its ABC transporter, encoded by the *mel* operon, were responsible for the uptake of the raffinose family of oligosaccharides, the most widespread d-galactose-containing oligosaccharides in higher plants. Hence, the presence of the *mel* operon in the core genome of *A. fabrum* indicates the adaptation of this species to plant-related niches.

### Conclusions.

We focused our analysis on *A. fabrum* due to its well-known biotechnological applications, well-sequenced genomes, unusual chromosomal structure, diverse ecological niches, and poorly understood genetic diversity and evolutionary dynamics. Here, we conducted comprehensive pan-genome analysis of the *A. fabrum* chromosome using 25 genomes, selected by core genome phylogeny, in combination with the ANI, AAI, and *in silico* DDH values. The open pan-genome exhibits high genetic diversity with a flexible gene repertoire within the accessory genome and strain-specific genes, which promotes genomic evolution. This result is more prominently reflected by the pan-genome constructed from the representative genomes. *A. fabrum* genomes exhibit a high level of genetic plasticity characterized by tRNA loci, diverse MGEs with heterogeneous distribution, a small number of barriers to HGT, and potential horizontal genes, potentially contributing to the expansion of gene pools for niche adaptation. Comparative analysis revealed significant differences in the functional enrichment and degree of purifying selection between the core and non-core genomes. The core genome content is more conserved than that of the accessory genome, indicating a stronger tendency to maintain its functions. Despite undergoing stronger purifying selection, core gene families have a significant bias to contain one or more codon sites under positive selection as being evolutionary significant. These results may indicate that the core gene families are constrained by stronger purifying selection due to their importance for the species’ basic function, with a few variations permitted to promote gentle adaptation to diverse niches. Additionally, potential variations in metabolic properties driven by positive selection were observed, reflecting that the adaptation of *A. fabrum* to ecological niches is promoted by adaptive mutations. Our genomic analysis also revealed diverse CAZymes in *A. fabrum*, indicating that the existence of CAZymes in the accessory and strain-specific genomes may contribute to adaptation to diverse environments, particularly the rhizosphere. Numerous potential virulence genes are present in the chromosome. Examination of potential virulence genes in the genetically tractable *A. fabrum* may also serve to elucidate the molecular roles of these genes in pathogenicity. Detailed pan-genome analysis of *A. fabrum* provides useful understanding of its genetic diversity, evolutionary dynamics, and niche adaptation, while the detailed elucidation of how genomic features function requires further biological studies.

Our study also provides a wealth of comparative data for greater understanding of the genetic diversity and evolutionary dynamics between the *A. fabrum* Chr I and chromid from the pan-genome perspective. Although previous studies have found that the chromid of the C58 genome exhibited higher plasticity and was much less conserved than the Chr I, our results demonstrate the Chr I and chromid pan-genomes represent similar genetic diversity. Both Chr I and chromid possess diverse MGEs and horizontal genes, which correspond to similar genetic diversity between these two chromosomes. Next, we utilized a comparative genomics approach to analyze the functional and evolutionary divergence between Chr I and the chromid. Significant differences in the prevalence of COG functional categories were observed in each component of the pan-genome between Chr I and the chromid. We observed a special evolutionary pattern on each chromosome in *A. fabrum*. Overall, the Chr I gene families have experienced a similar degree of purifying selective pressure as the chromid gene families. Notably, significant differences in evolutionary constraints were observed between Chr I and chromid in the core and accessory genome, respectively. The Chr I core gene families have undergone significantly stronger purifying selection than those of chromid. In contrast, the chromid accessory gene families are significantly more strongly preserved and evolve more slowly than those of Chr I. This model is not applicable to other multi-chromosomal bacteria (e.g., *Vibrio*, *Burkholderia*, and Sinorhizobium meliloti). Hence, future pan-genome analyses of other species with these features will help to elucidate the evolutionary role of multiple chromosomes in bacteria.

## MATERIALS AND METHODS

### Genome collection and analysis.

All available sequenced *A. fabrum* genomes were collected and defined by the taxonomically united genome database in EzBioCloud (39) (www.ezbiocloud.net/) and NCBI GenBank (www.ncbi.nlm.nih.gov/genbank/) (Table S1; last accessed 30 April 2022). The collection contained 28 genomes, including 7 complete and 21 draft genomes. The genome sequence for *A. fabrum* S2_009_000_R2_73 was found to be incomplete and was thus eliminated from further data processing. The remaining 27 genomes were estimated to be 99.9 ± 0.4% complete with 0.1 ± 0.3% contamination using CheckM v1.0.13 (40). Two of the *A. fabrum* genome sequences found in public databases, DE0067 (accession no. GCA_007679885.1) and DE0068 (accession no. GCA_007679845.1), failed the taxonomy verification in the NCBI GenBank pipeline (41). The genome sequences derived from *A. fabrum* PDC82, NFIX01, NFIX02, AFS036755, AFS086441, and LBA4404 were reclassified according to the EzBioCloud genome database because they were originally identified as *Rhizobium* sp., *Hyphomicrobiales*, or A. tumefaciens. Eleven reference genomes of closely related *Agrobacterium* species were collected in combination with 27 *A. fabrum* genomes for subsequent analyses. A detailed account of the collected genomes, including strain names, accession numbers, taxonomy, assembly type, genomic length, GC content, completeness, and contamination is shown in Table S1. Unified gene finding and re-annotation of the *Agrobacterium* spp. genomes were performed based on the RAST server (https://rast.nmpdr.org/) (42). Scaffolds and contigs in the draft genomes of *A. fabrum* (*n *=* *18) were aligned to the chromosomes of the reference complete genome (C58; GCA_000092025.1) using Mauve Genome Alignment software v2.4.0 (43). The scaffolds or contigs were mapped to the reference genome to determine their locations (Chr I, AE007869.2; circular chromosome or chromid, AE007870.2; linear chromosome), with a cutoff of 60% coverage. The gene families located in chromosomes were used to perform the pan-genome analysis.

### Pan-genome analysis.

Orthologous groups of protein families of pan-genome were delimited using OrthoFinder2 software with the DIAMOND method (44, 45). The OrthoFinder output files (Orthogroup_Sequences folder) were used to extract pan-genome families (total of all genes found across strains), core genome families (genes shared among all strains), accessory genome families (genes shared among more than one strain, but not in all), and strain-specific genes (genes found only in one strain). Curve-fitting of the pan-genome was performed using a power-law regression based on Heaps’ law ($n=\kappa N^{\gamma }$) (16, 46), where *N* is the number of genomes, κ is a proportionality constant, and a growth exponent of *γ* > 0 indicates an open pan-genome. A descriptive statistical analysis was generated using OriginPro 9 software with the Allometric1 model.

### Phylogenetic analysis.

The core genome phylogenetic analysis was performed based on single-nucleotide polymorphisms (SNPs) across single-copy core gene families extracted from the OrthoFinder output files. Nucleotide sequences of the single-copy core gene families (*n *=* *2,252) were extracted according to the protein accession numbers and then aligned using the MAFFT v7.508 software (47). The set of SNPs present in single-copy core gene families was extracted and then integrated according to the arrangement of the genes on the *A. fabrum* C58 genome (complete genome). To avoid phylogenetic confusion, we identified and removed the putative recombinational regions from the SNP set using ClonalFrameML v1.12 software (48). A maximum likelihood tree was constructed using MEGA 7 (49) with the general time-reversible model and 100 bootstrap replicates.

### Comparative genomic analysis.

The program pyani v0.2.x based on the MUMmer method (ANIm) (50, 51) CompareM (https://github.com/dparks1134/CompareM) was used to calculate the average nucleotide identity and amino acid identity. The *in silico* DNA-DNA hybridization value was calculated using the online interface of the Genome-to-Genome Distance Calculator v2.1 (14). The prophages were predicted using the online interface of PHAge Search Tool—Enhanced Release (PHASTER) (52). The online interface of IslandViewer 4 (53) (integrating three different methods: SIGI-HMM [54], IslandPath-DIMOB [55], and IslandPick [56]) was utilized to identify genomic islands. ISs were predicted using the online interface of ISfinder (57). The CRISPR and *cas* genes were predicted using the CRISPRCasFinder v4.2.2 with default parameters (58). The gene families of the pan-genome were functionally characterized by COG functional category (59) using eggNOG-mapper v2.1.9 software (60). The gene cluster related to secondary metabolism was identified and analyzed using antiSMASH 6.1.1 (34) with the default parameters. The genes encoding carbohydrate binding and metabolic enzymes were identified using the dbCAN2 database (33) by an HMMER search (61).

### Identification of potential horizontal genes.

HGTector v2.0 (62) with the database 2021-11-21 was used to identify potential horizontal genes in *A. fabrum*. The *A. fabrum* (rank: species; taxon ID: 1176649) and *Rhizobiaceae* (rank: family; taxon ID: 82115) were set as self-group and close-group, respectively.

### Pressure selection analysis.

Positive selection in coding regions can be estimated by calculating the ratio of the nonsynonymous substitution rate to the synonymous substitution rate (*dN*/*dS*). ParaAT v2.0 software was used for codon-based alignment of the orthologous genes (63), and the Fast Unconstrained Bayesian Approximation (FUBAR) pipeline (64) of HYPHY v2.5.42 software was used to measure the *dN*/*dS* ratio at each site in each orthologous gene family. The codon sites subject to diversifying positive selection were inferred at a posterior probability of ≥0.9.

### Identification of the genotypic and phenotypic profiles of virulence genes.

To identify the virulence factors, the protein sequences of all genomes were aligned using BLASTp with E value cutoff < 1e-6, identity > 60%, and coverage > 60% against the data set from the Pathogen Host Interactions database (PHI-base v5.0) (35). Results were visualized using the pheatmap package in R.

### Identification of the key genomic elements.

To examine *agp* gene, *mel* operon, and SpG8-1a to -7b, we curated the reference genes implicated in previous studies (12, 36, 37) to screen using the LS-BSR v3.0 tool with default parameters (65). A gene with a BSR score of <0.6 was considered absent. Results were visualized using the pheatmap package in R.
