# Supplementary material for: Pan-Chromosome and Comparative Analysis of Agrobacterium fabrum Reveal Important Traits Concerning the Genetic Diversity, Evolutionary Dynamics, and Niche Adaptation of the Species
Source: Microbiol Spectr. 2023 Feb 28;11(2):e02924-22. doi: 10.1128/spectrum.02924-22 (PMC10100860; doi:10.1128/spectrum.02924-22)
Supplement: Supplemental file 1 — Fig. S1 to S3. Download spectrum.02924-22-s0001.pdf, PDF file, 0.4 MB [file spectrum.02924-22-s0001.pdf]

SUPPLEMENTAL MATERIAL FIGURE

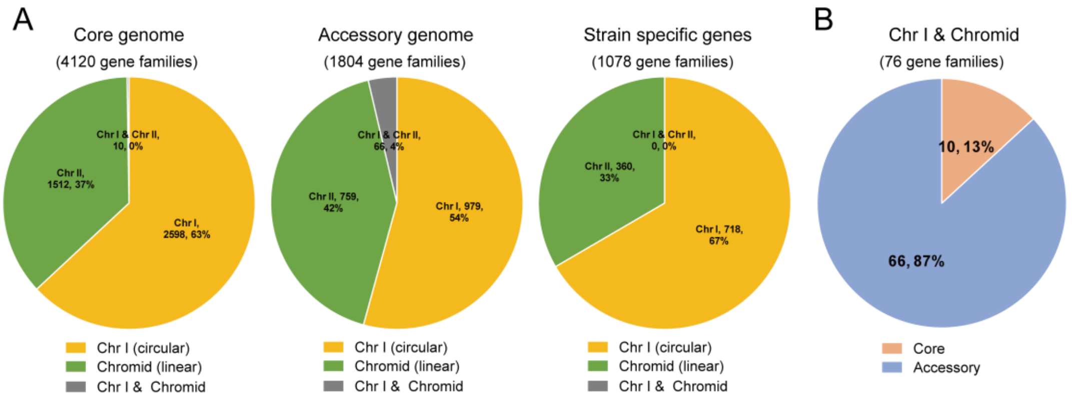

Supplementary FIG S1 The components of pan-genome distributed in the chromosomes. A. Distribution of the core, accessory, and strain-specific gene families in the chromosomes, respectively. B. Pie charts represent the percentage of the core, accessory, and strain-specific gene families participating in the gene families shared by both chromosomes.

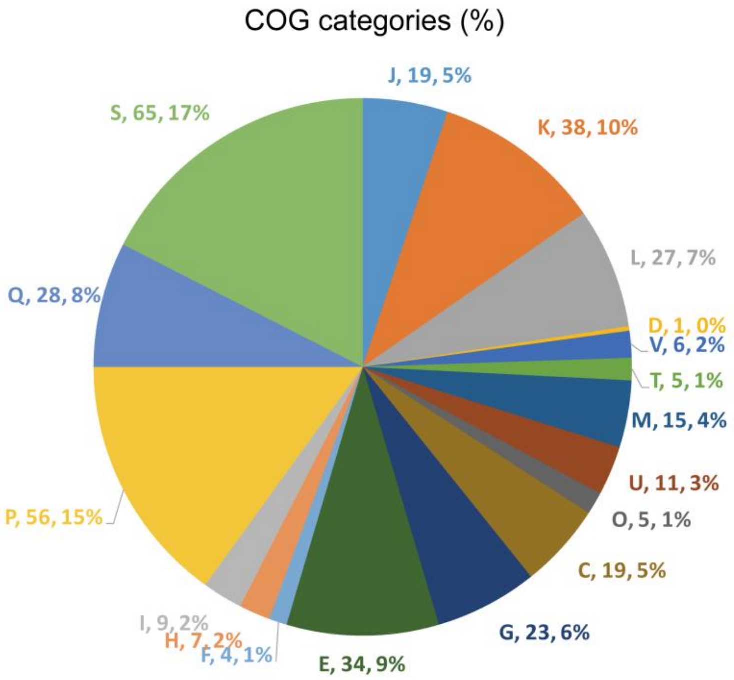

Supplementary FIG S2 Distribution of Clusters of Orthologous Groups (COG) functional categories for the potential horizontal gene families.

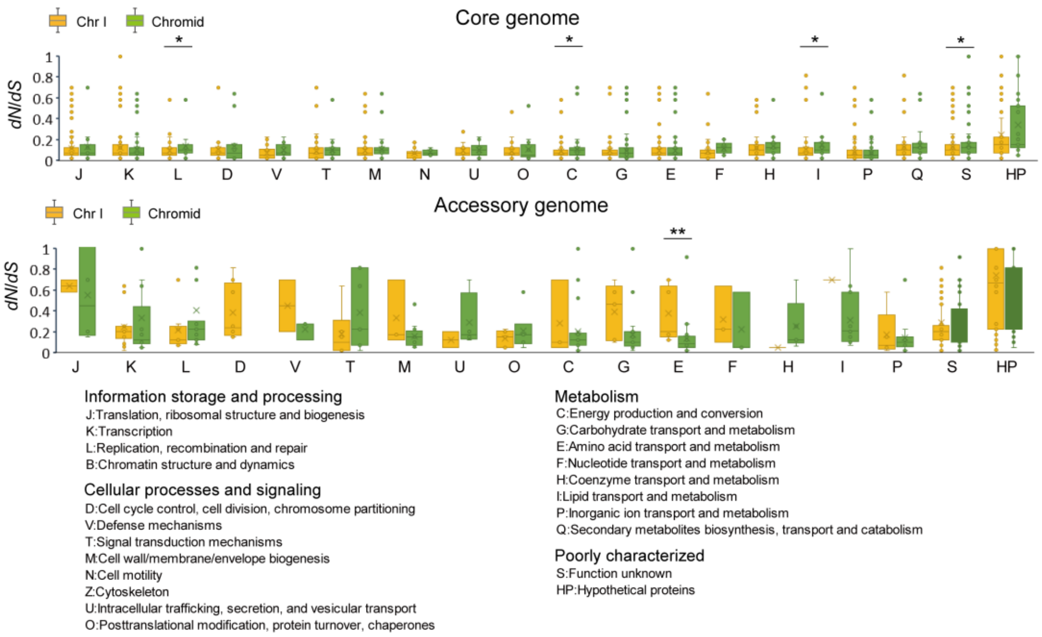

Supplementary FIG S3 Comparisons of the dN/dS rates of core gene families and accessory gene families between Chr I and chromid in COG functional categories. *t-test P-value < 0.05; **t-test P-value < 0.01.
